# The use of machine learning modeling, virtual screening, molecular docking, and molecular dynamics simulations to identify potential VEGFR2 kinase inhibitors

**DOI:** 10.1038/s41598-022-22992-6

**Published:** 2022-11-05

**Authors:** Abbas Salimi, Jong Hyeon Lim, Jee Hwan Jang, Jin Yong Lee

**Affiliations:** 1 Department of Chemistry, Sungkyunkwan University, Suwon, 16419 Korea; 2 School of Materials Science and Engineering, Sungkyunkwan University, Suwon, 16419 Korea; 3 Ucaretron Inc., No. 3508, 40, Simin-daero 365 beon-gil, Dongan-gu, Anyang-si, Gyeonggi-do Korea

**Keywords:** Drug discovery, Virtual screening

## Abstract

Targeting the signaling pathway of the Vascular endothelial growth factor receptor-2 is a promising approach that has drawn attention in the quest to develop novel anti-cancer drugs and cardiovascular disease treatments. We construct a screening pipeline using machine learning classification integrated with similarity checks of approved drugs to find new inhibitors. The statistical metrics reveal that the random forest approach has slightly better performance. By further similarity screening against several approved drugs, two candidates are selected. Analysis of absorption, distribution, metabolism, excretion, and toxicity, along with molecular docking and dynamics are performed for the two candidates with regorafenib as a reference. The binding energies of molecule1, molecule2, and regorafenib are − 89.1, − 95.3, and − 87.4 (kJ/mol), respectively which suggest candidate compounds have strong binding to the target. Meanwhile, the median lethal dose and maximum tolerated dose for regorafenib, molecule1, and molecule2 are predicted to be 800, 1600, and 393 mg/kg, and 0.257, 0.527, and 0.428 log mg/kg/day, respectively. Also, the inhibitory activity of these compounds is predicted to be 7.23 and 7.31, which is comparable with the activity of pazopanib and sorafenib drugs. In light of these findings, the two compounds could be further investigated as potential candidates for anti-angiogenesis therapy.

## Introduction

Increasing prevalence of cancer extremely intimidating to human health and causes death worldwide. Despite the huge advances in cancer therapy, its mortality is still high due to phenotypic diversification and its complex genetic makeup^1^. Therefore, it is necessary to discover and develop novel anticancer drugs. Angiogenesis and vasculogenesis are the complicated processes of the formation of new capillaries from pre-existing blood vessels that under normal conditions are pivotal to maintaining nutrients and oxygen for cellular proliferation, tissue repair, pregnancy, and normal embryogenesis^2–4^. Abnormal angiogenesis, which is known as neo-angiogenesis, leads to numerous pathological disorders, such as rheumatoid arthritis, inflammation, psoriasis, retinal diseases, and particularly the growth and metastasis of a variety of cancers, such as lung, colon, and kidney cancer as well as glioblastoma^4–6^. Many types of research have studied regulating angiogenesis to combat cancer. Vascular endothelial growth factor (VEGF) is the main regulator of the angiogenesis process^6,7^.

The overexpression of epidermal growth factor receptor (EGFR) can be observed in different types of cancer. In a pathological pathway, activation of EGFR can enhance the expression of VEGF^8^. VEGF can trigger angiogenesis by binding to the vascular endothelial growth factor receptor (VEGFR) and signaling via VEGFRs. These transmembrane proteins, which are known as tyrosine kinases, include VEGFR1(Flt-1), VEGFR2(Flk-1/KDR), and VEGFR3(Flt-4), and are presented on the surface of endothelial and lymphatic cells^5,6,8,9^. Among these receptor protein tyrosine kinases, VEGFR2 is the main effector of VEGF-mediated cell proliferation and plays a critical role in the angiogenesis of a tumor. The VEGFR2 structure is a type III transmembrane kinase receptor composed of the extracellular part, a short transmembrane domain, and a tyrosine kinase domain^10,11^. VEGF/VEGFR2 binding results in downstream signaling activation. Thus, VEGFR2 is highly autophosphorylated during the growth of a tumor^5,12^. Due to its role, VEGFR2 has been identified as a therapeutic target for the design of novel inhibitors to hinder angiogenesis^2,3,8,13^. As a result, inhibition of the VEGFR2 signaling pathway has been considered a major, attractive target for anti-angiogenesis therapy for the treatment of cancer^5,6,12,14^.

**Figure 1:**
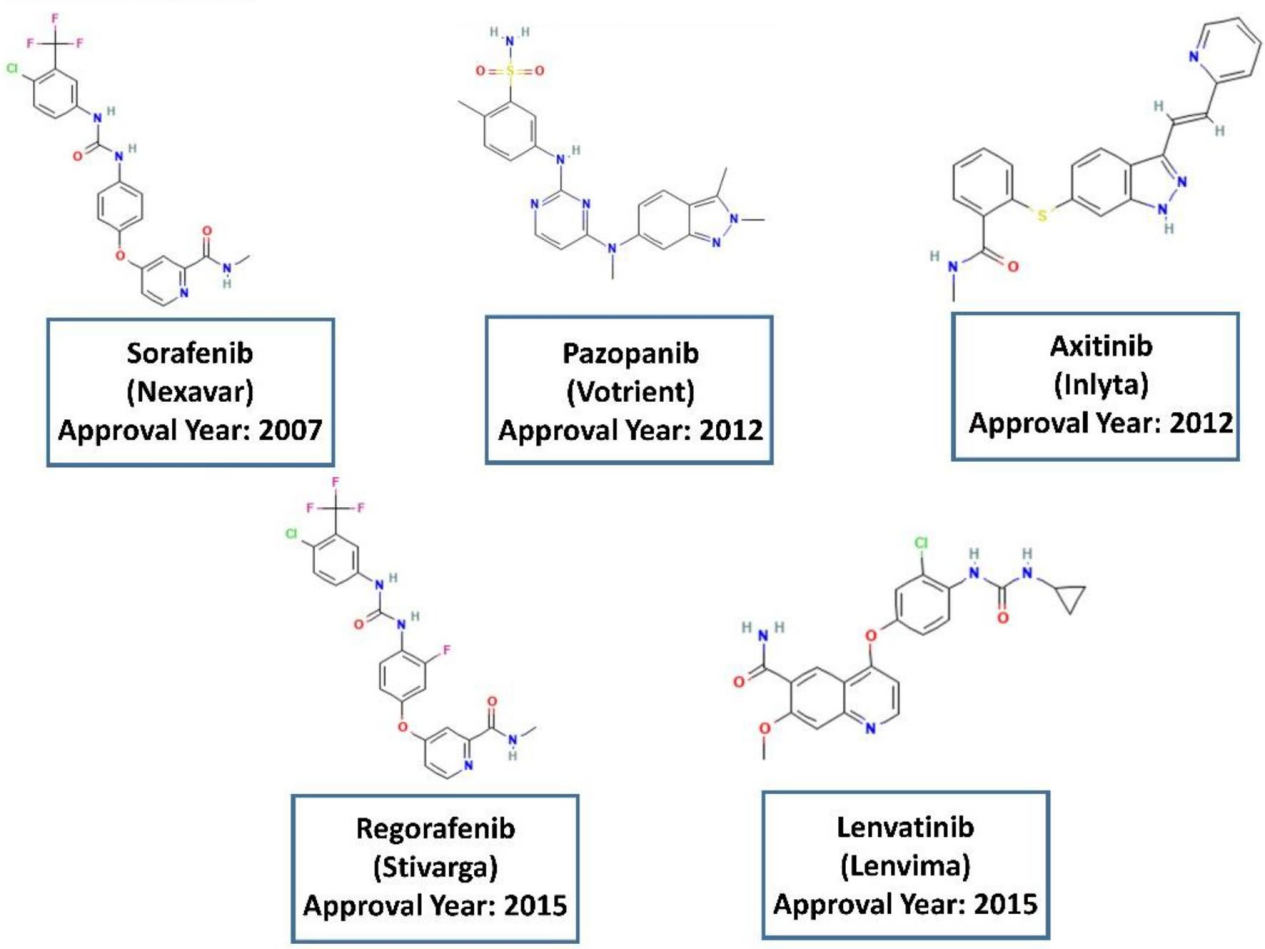
Some of the clinically approved VEGFR2-kinase inhibitors.

To date, several kinds of drugs, such as sorafenib, pazopanib, axitinib, regorafenib, and lenvatinib, have been approved by Food and Drug Administration (FDA) for VEGFR2 inhibition^1,11^. Some of these clinically approved VEGFR2 inhibitors are depicted in Fig. 1. The use of anticancer drugs has been accompanied by side effects and drug resistance that can reduce the efficacy of the cancer treatment^1,11^. Thus, it is highly desirable to discover and develop novel drugs, ideally with fewer side effects and improved efficacy, but it is a very complicated and challenging issue^1,11,12^. Quantitative structure–activity relationship (QSAR) modeling is an important method for drug discovery that indicates correlations between the molecular structure to biological properties and activities using molecular descriptors^15,16^. Virtual screening is an in silico method for screening a large number of molecules to avoid the cost of experimental examinations to find potential bioactive candidates. This effective tool has been used as a popular approach for drug discovery^17^. Different types of studies with a variety of methods and approaches have been used in virtual screening^18–20^. The screening can be ligand- or structure-based. The ligand-based screening can be done using fingerprint similarity, shape-based similarity, or machine learning (ML) methods^17^. Several models, such as artificial neural network (ANN), random forest (RF), naïve Bayesian (NB), and support vector machine (SVM), have been applied to classify compounds based on their activity^1^. Similarity searching techniques are important methods to find molecules that share similar bioactivity in pharmaceutical research and drug discovery^21^. One of the most common ways to compare the similarity between molecules is to convert the structure of the molecules into bit vectors. The standard quantification method for similarity searching using bits is the Tanimoto coefficient^17^. There are various types of fingerprints, such as MACCS, usually with 166 bits, PubChem fingerprints with 881 bits, BCI fingerprints with 1052 bits, and circular fingerprints like Extended-Connectivity Fingerprints (ECFPs) that are based on the Morgan algorithm with different radii and bit vectors^17^. Generally, longer bit vectors of fingerprints can result in better performance^22^. Using the fingerprint calculation for an active compound in a dataset and applying the similarity coefficient we can rank the molecules to determine the molecules with the higher similarity to the reference molecule^17^. The performance of the screening methods sometimes may depend on the chosen target^17^ and in many cases, the methods based on the 2D fingerprints can outperform approaches based on a 3D shape^23,24^. Virtual screening based on fingerprints is less CPU-intensive and can be done with less progression. Widely used fingerprints are primarily obtained from 2D structures. However, there are some issues in similarity searching based on fingerprints, such as choosing the proper fingerprints and the activity of the reference molecule^17,23–25^. Machine learning and computational approaches have been applied in research to predict VEGFR2 inhibitors. For instance, De Kang et al. have reported on discovering VEGFR2 inhibitors using naïve Bayesian classification, docking evaluation, and drug screening methods^1^. Sobhy et al. have employed 3D-QSAR pharmacophore modeling in combination with virtual pubchemscreening and docking characterization to find a novel scaffold to inhibit VEGFR2^26^. Zhang et al. have conducted an integrated virtual screening method and molecular docking estimation for targeting VEGFR2^27^.

**Figure 2:**
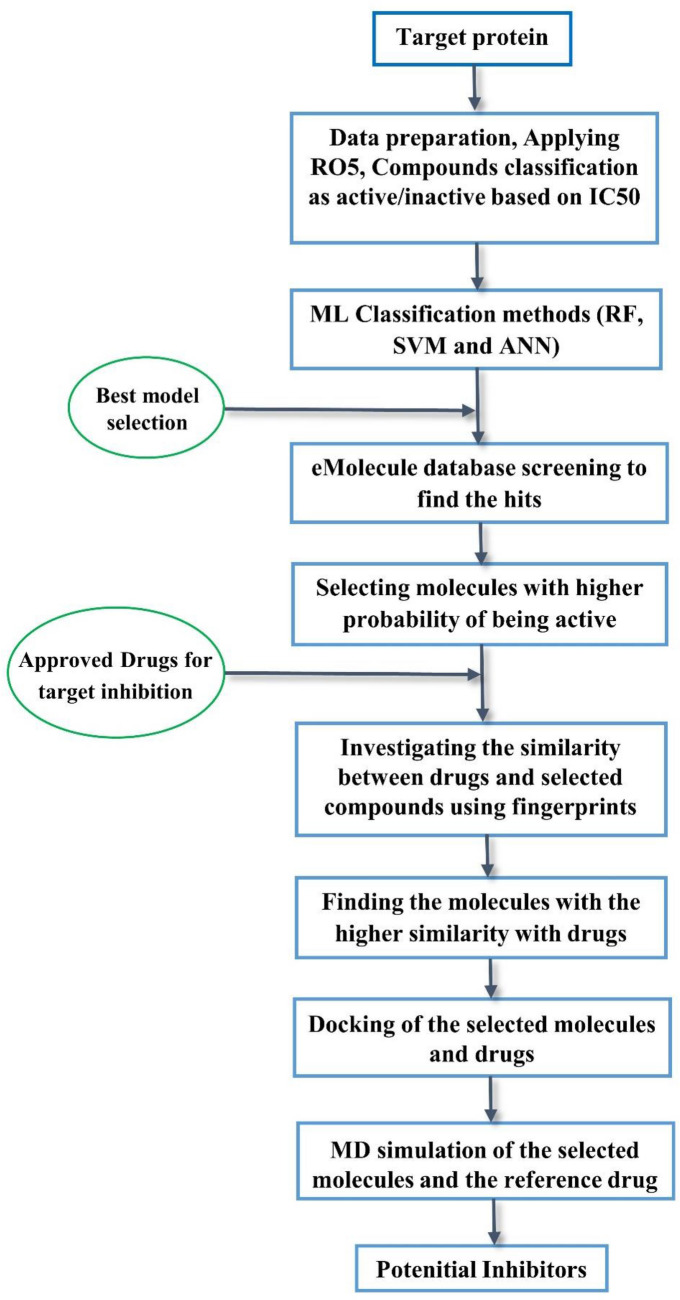
Detailed workflow of the study to find potential inhibitors.

In the current research, we constructed a workflow based on machine learning models to screen a large number of molecules (~ 10,500,000) for potential novel inhibitors of VEGFR2 that could be helpful to fight cancer. The detailed workflow is shown in Fig. 2. We applied cheminformatics, machine learning, predictions of absorption, distribution, metabolism, excretion, and toxicity (ADMET), molecular docking characterization, and molecular dynamics (MD) simulations to find new potential VEGFR2 inhibitors.

## Materials and methods

### Databases

The OpenCADD^28^ platform as an open-source for cheminformatics was used for the acquisition of compound data and the generation of machine learning models. Information on the bioactivities of the related compounds was collected using the ChEMBL database^29^. Also, more than 10 million molecules from eMolecules databases (http://emolecules.com/) were taken as an external library for virtual screening.

### Evaluation metrics of predictive models

To evaluate the classifier models, accuracy, sensitivity, specificity, and AUC are calculated to assess the models' performance. In these metrics, true-positive (TP) is the number of inhibitors that are correctly determined as active compounds while the true-negative (TN) is the number of decoys that are correctly defined as inactive compounds. False-positive (FP) indicates the number of decoys that are classified as active even though they are not active. The number of inhibitors that are incorrectly classified as inactive compounds was defined as false-negative (FN). The AUC measures the true positive rate (TPR) versus the false positive rate (FPR) to evaluate the classification models. To measure the accuracy of the regression model correlation coefficient (R2), mean absolute error (MAE), and root-mean-squared error (RMSE) were calculated as below.

$$\begin{aligned} & {\text{Accuracy }} = \, \left( {{\text{TP}} + {\text{TN}}} \right)/\left( {{\text{TP}} + {\text{FP}} + {\text{FN}} + {\text{TN}}} \right) \\ & {\text{Sensitivity }} = {\text{ TP}}/\left( {{\text{TP}} + {\text{FN}}} \right) \\ & {\text{Specificity }} = {\text{ TN}}/\left( {{\text{TN}} + {\text{FP}}} \right) \\ \end{aligned}$$

TP = True Positive, FP = False Positive, TN = True Negative, FN = False Negative

$$\begin{aligned} & {\text{MAE}} = \frac{1}{{\varvec{N}}}\mathop \sum \limits_{{{\varvec{i}} = 1}}^{{\varvec{N}}} \left| {{\varvec{y}}_{{\varvec{i}}} - \hat{\user2{y}}} \right| \\ & {\text{RMSE}} = \user2{ }\frac{1}{{\varvec{N}}}\sqrt {\mathop \sum \limits_{{{\varvec{i}} = 1}}^{{\varvec{N}}} \left( {{\varvec{y}}_{{\varvec{i}}} - \hat{\user2{y}}} \right.)^{2} } \\ & {\text{R}}^{{2}} = { 1} - \frac{{\mathop \sum \nolimits_{{{\varvec{i}} = 1}}^{{\varvec{N}}} \left( {{\varvec{y}}_{{\varvec{i}}} } \right. - \hat{\user2{y}})^{2} }}{{\mathop \sum \nolimits_{{{\varvec{i}} = 1}}^{{\varvec{N}}} \left( {{\varvec{y}}_{{\varvec{i}}} } \right. - \overline{\user2{y}})^{2} }} \\ \end{aligned}$$

where, $\overline{{\varvec{y}} }$ and $\widehat{{\varvec{y}}}$ represent the predicted value of y and the mean value, respectively.

### Molecular docking

The 3D structure of VEGFR2 for docking was retrieved from the protein data bank (PDB: 4ASE). Docking was performed on seven known drugs (pazopanib, sorafenib, axitinib, regorafenib, lenvatinib, motesanib, and Ki8751) and the two candidate compounds (molecule1 (PubChem CID 17379777 and molecule2 (PubChem CID 4682044)) by the AutoDock Vina in UCSF Chimera v.1.12 software^30^ to estimate the binding affinities and find best-scored poses for MD simulation. The Dock prep was performed which includes deleting the solvent and adding hydrogens and charges. To assign charges for the standard residues AMBER ff14SB and other residues, Gasteiger has been selected that compute charges using ANTECHAMBER. We defined the receptor search volume size based on the possible binding pocket which was around 20 Å in each direction. The number of binding modes, exhaustiveness of search, and maximum energy difference (kcal/mol) was set at 10, 8, and 3, respectively. The docked poses with the highest score were selected for further MD calculations.

### Molecular dynamics (MD) simulation

The best scored-pose of regorafenib, molecule1, and molecule2 docked with VEGFR2 were refined using MD simulations. The CHARMM 36 forcefield and CGenFF parameters^31^ were used to calculate the ligand–protein interactions. All MD simulations were conducted in a cubic grid box at a constant temperature of 300 K and a constant pressure of 1 atm. Long-range electrostatic interactions were calculated using the particle-mesh Ewald method. The temperature was controlled by the V-rescale method^32^. The systems were solvated using TIP3P water molecules. The equilibration steps were applied on NVT following the NPT for 100 ps. The MD simulation was performed with a 2 fs time step using the Parrinello-Rahman method^33^. The MD simulations were performed in triplicates. By calculating the RMSD, the stability of the complexes was accessed and the well-converged part was used for binding free energy analysis.

### Bioactivity and toxicity

In drug discovery, we need to consider the lead-likeness of the compounds. In this respect, Lipinski’s Rule of Five (RO5), also known as Pfizer's rule^34^, was applied to screen the data based on molecular weight (MW) less than 500 Da, number of hydrogen bond acceptors (HBAs) **≤** **10,** number of hydrogen bond donors (HBDs) ≤ 5, and octanol–water coefficient (LogP) ≤ 5. RO5 provided some simple criteria in the initial stages of drug discovery to examine whether a chemical had the properties to be like an orally-taken drug^35,36^. We estimated the absorption or solubility of those compounds. In addition, compounds were subjected to screening against unwanted substructures and Pan Assay Interference Molecules (PAINS) using an RDKit^27^.

For unwanted groups that could cause toxicity the compounds with mutagenic functionalities, such as nitro and phosphate groups and reactive groups like 2‐halopyridines, were removed^37^. The use of PAINS could cause false positives in biological screening. Some of those, such as catechols and rhodanines, could cause artifacts by representing a high activity^38^. Baell et al. defined 480 substructures as bioassay interference that could be involved in interaction with non-specific targets^39^. Roughly 5% of drugs approved by the FDA include PAINS substructures^40^.

## Results and discussion

### Machine learning classification models

**Figure 3:**
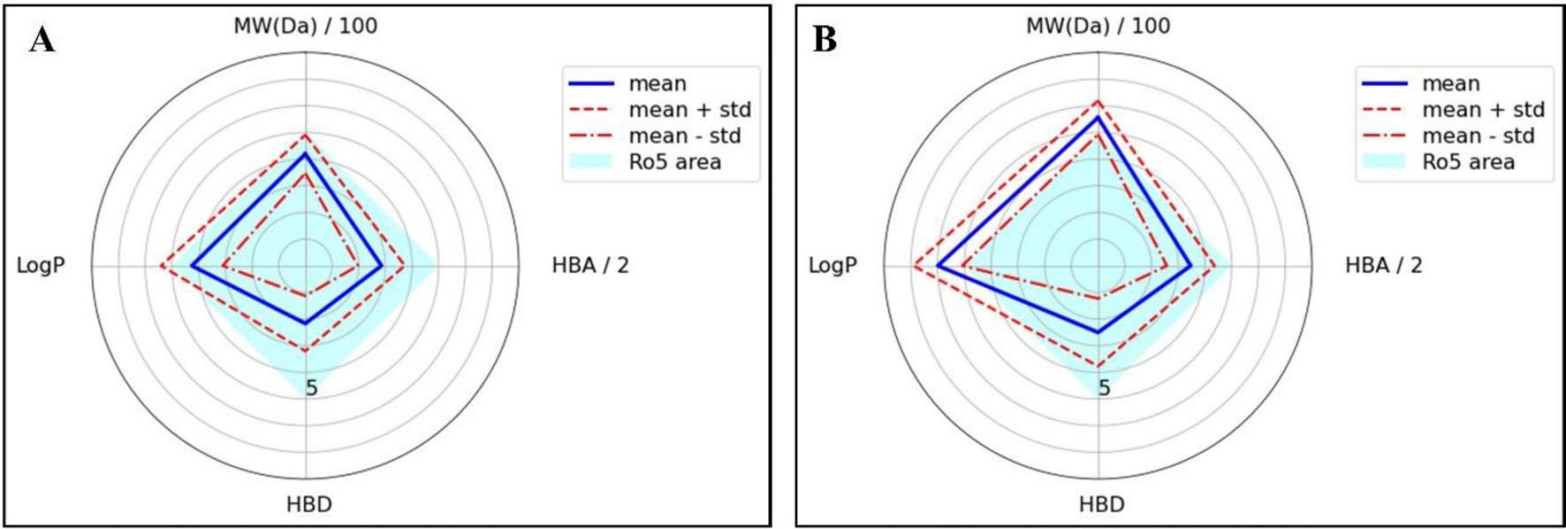
Physio-chemical radar plots of the compounds in the dataset that (**A**) fulfill the Rule of Five (RO5) or (**B**) violate the RO5. Abbreviations: molecular weight (MW), hydrogen bond acceptors (HBA), number of hydrogen bond donors (HBD), and partition coefficient (LogP). The figure is created by using scikit-learn v 0.23.1, https://scikit-learn.org.

To perform QSAR classification, three supervised machine learning classifiers, including ANN^41^, RF, a popular method with good performance in QSAR^42,43^, and SVM^44^ using the TeachOpenCADD pipeline were employed. The models were built using the Morgan3 protocol with 2048 bits as a circular fingerprint that was implemented in the RDKit toolkit^27^ to encode the molecules. A total of 7050 data (after cleaning the initial 8572 data) for VEGFR2 inhibitors from the ChEMBL database was used to generate the models. After applying the RO5, a total of 5789 data that fulfilled the Lipinski rule remained. The radar or spider plots of physicochemical properties of the dataset’s compounds that fulfilled or violated the RO5 are shown in Fig. 3. The cyan square displayed the area where the data parameters met the RO5 conditions. The blue and the red dashed lines provide the mean and standard deviations, respectively. Figure 3A shows that there was no violation of mean values, but the standard deviation showed slightly larger values than the RO5 criteria for some properties. This was acceptable because, according to the RO5, one of the four rules can be violated^45^. It can be seen in Fig. 3B that some violations happened in MW and LogP values.

The pIC50 cut-off value was set at 6.8 to define the active and inactive binary labels as a cut-off ranging from the value of 5 to 7 has been suggested, which resulted in 2978 active and 2811 inactive compounds. The dataset was randomly split into a training/test (80/20) set. The classifiers were generated by Python library Scikit-learn^46^ and the hyper-parameters of each model, such as the number of trees, SVM kernel, gamma, and hidden layers, were adjusted to achieve the best performance. To examine the model performance, the assessment measures including sensitivity, specificity, accuracy, and area under the curve (AUC) of the receiver operating characteristic (ROC) were calculated on the test set and shown in Table 1.

**Table 1:** Evaluation of machine learning models applied to the VEGFR-2 data.

Models	Sensitivity	Specificity	AUC	Accuracy
RF	0.85	0.82	0.91	0.83
SVM	0.83	0.83	0.91	0.83
ANN	0.81	0.78	0.87	0.80

Also, fivefold cross-validation (CV) runs with random selections of 20% of the data were evaluated (Table 2).

**Table 2:** Model validation using cross-validation on VEGFR-2 data.

Models	Sensitivity	Specificity	AUC	Accuracy
RF	0.84 (± 0.01)	0.81 (± 0.01)	0.90 (± 0.01)	0.83 (± 0.01)
SVM	0.83 (± 0.02)	0.82 (± 0.01)	0.90 (± 0.01)	0.83 (± 0.01)
ANN	0.80 (± 0.02)	0.79 (± 0.01)	0.87(± 0.01)	0.79 (± 0.01)

**Figure 4:**
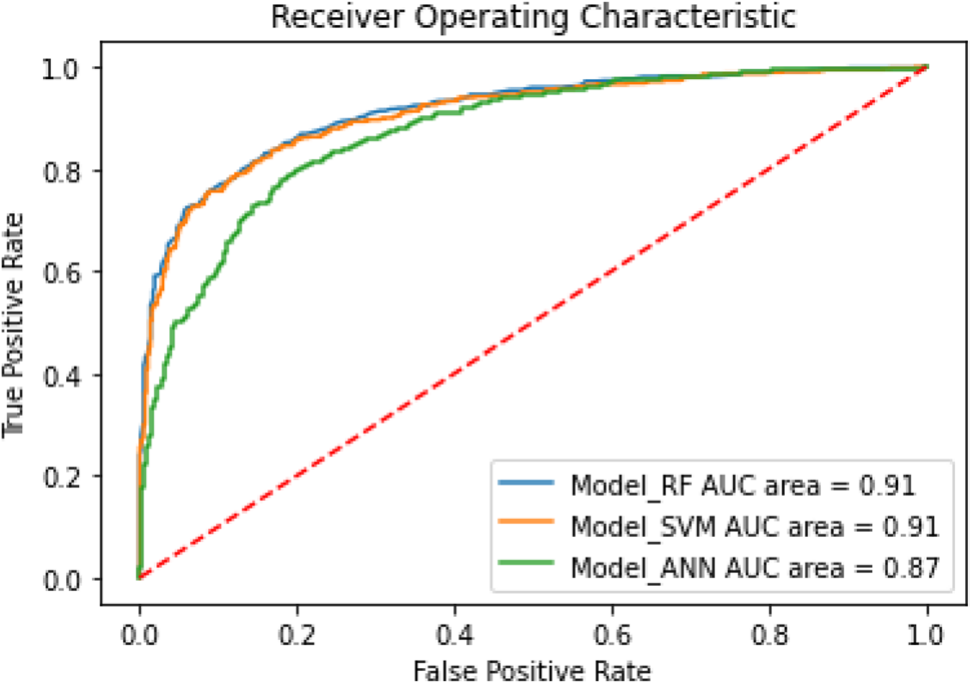
Receiver operating characteristic plot. The figure is created by using scikit-learn v 0.23.1, https://scikit-learn.org.

Based on the model's AUC values (0.91, 0.91, and 0.87, Fig. 4), it appeared that our models could predict classification well. The RF and SVM models with higher AUC values showed slightly better performance than the ANN system. To predict the activity of the compounds in the eMolecules database (~ 10,500,000 compounds) the RF model was selected for virtual screening due to its slightly higher sensitivity compared to the SVM model. After screening to sample the most appropriate compounds, the molecules with a higher predicted probability (more than 85%) were selected and filtered against the PAINS and unwanted substructures. In this manner, we determined 66 chemical compounds as likely to be potential active inhibitors. After final filtration, 61 compounds remained.

### Similarity measures

Computational similarity measures have been used widely in drug design and chemical informatics despite some limitations, including the assumption for virtual screening that compounds with similarities in structure may show similar biological activities^47–49^. Previous studies using statistical analysis have revealed the reliability of Tanimoto and Dice indexes to quantify the molecular similarity as comparison metrics^50^. In the next step of our workflow, tanimioto_maccs, dice_maccs, tanimoto_morgan, and dice_morgan similarity metrics are used to calculate the chemical similarity between those 61 compounds and some selected known inhibitors by converting the structures into bit vectors^51^. Similarity checking is performed for pazopanib, sorafenib, axitinib, regorafenib, lenvatinib, motesanib, and Ki8751 by applying the Morgan2 and MACCS fingerprints (Supplementary Figure S1). After performing the similarity measure with the top-ranked molecules, the top 4 compounds with the highest similarity with each reference considering Tanimoto_morgan, Dice_morgan, Tanimoto_maccs and Dice_maccs have been mentioned in Table 3. To obtain just a few possible candidates among those similar compounds, two structures (molecule1 (PubChem CID 17379777 and molecule2 (PubChem CID 4682044)) with the higher similarity indexes that happen more frequently as well are chosen for further analysis, including docking estimation and MD simulations.

**Table 3:** Similarity checking between Pazopanib, Sorafenib, Axitinib, Regorafenib, Lenvatinib, Motesanib, and Ki8751 with top-ranked molecules using the Morgan2 and MACCS fingerprints. (Compound 0 is the reference in each case).

Known inhibitors	Top 4 molecules	Tanimoto_morgan	Dice_morgan	Tanimoto_maccs	Dice_maccs
Pazopanib	0	1	1	1	1
	38	0.232877	0.377778	0.509804	0.675325
	23	0.226667	0.369565	0.750000	0.857143
	20	0.225352	0.367816	0.461538	0.631579
Sorafenib	0	1	1	1	1
	40	0.446154	0.617021	0.854167	0.921348
	16	0.433333	0.604651	0.729167	0.843373
	37	0.230769	0.375000	0.523077	0.686869
Axitinib	0	1	1	1	1
	38	0.232877	0.377778	0.509804	0.675325
	23	0.226667	0.369565	0.750000	0.857143
	20	0.225352	0.367816	0.461538	0.631579
Regorafenib	0	1	1	1	1
	40	0.369863	0.540000	0.854167	0.921348
	16	0.352941	0.521739	0.729167	0.843373
	56	0.230769	0.387755	0.580000	0.734177
Lenvatinib	0	1	1	1	1
	40	0.243902	0.392157	0.684211	0.812500
	16	0.236842	0.382979	0.636364	0.777778
	43	0.209877	0.346939	0.517857	0.682353
Motesanib	0	1	1	1	1
	46	0.305556	0.468085	0.744681	0.853659
	38	0.295775	0.456522	0.555556	0.714286
	42	0.260870	0.413793	0.510638	0.676056
Ki8751	0	1	1	1	1
	40	0.260274	0.413043	0.760000	0.863636
	56	0.250000	0.400000	0.7660000	0.863636
	55	0.235294	0.380952	0.530612	0.693333

**Figure 5:**
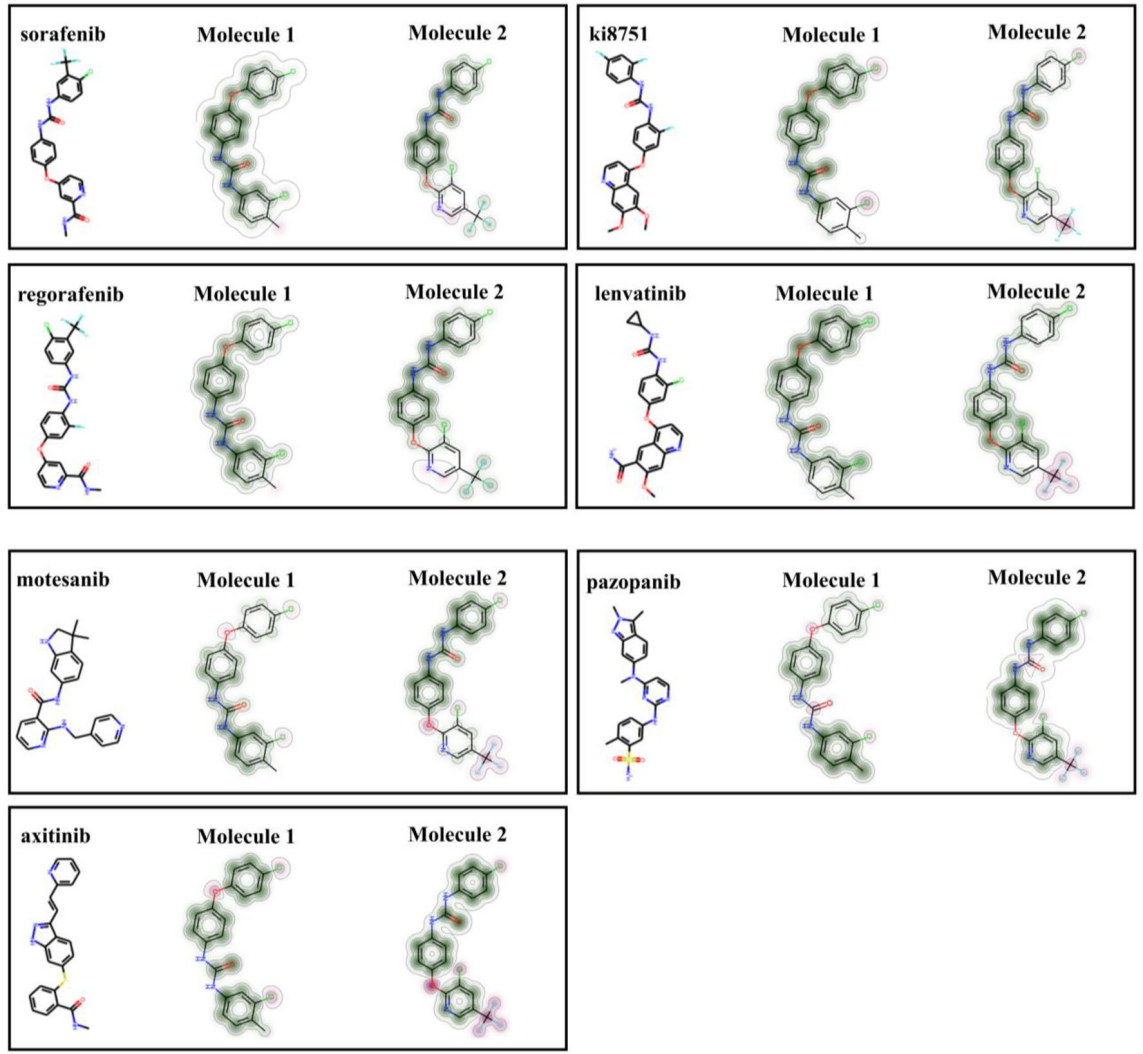
Similarity maps between sorafenib, Ki8751, regorafenib, lenvatinib, motesanib, pazopanib, and axitinib as references and candidates moelcule1 and molecule2 using the circular fingerprints (bit vector-based Morgan2). Coloring method: green: positive difference, gray: no change in similarity, and pink: negative difference. The figure is created by using Scikit-learn v 0.23.1, https://scikit-learn.org.

Using the Morgan2 fingerprint the similarity maps for these two compounds with seven known inhibitors as a reference can be formed, as shown in Fig. 5. This method can help visualize and compare the similarity between two molecules^27,51^. The green color indicates that removing bits will decrease the similarity, but the similarity increases by removing bits that have been shown in pink color. This type of comparison with the known inhibitors could provide us with more detailed information regarding the connection between the biological activities of the known drugs with the candidate compounds and how structural changes may increase or decrease the similarities.

### Prediction of pIC50 values using random forest regression

**Figure 6:**
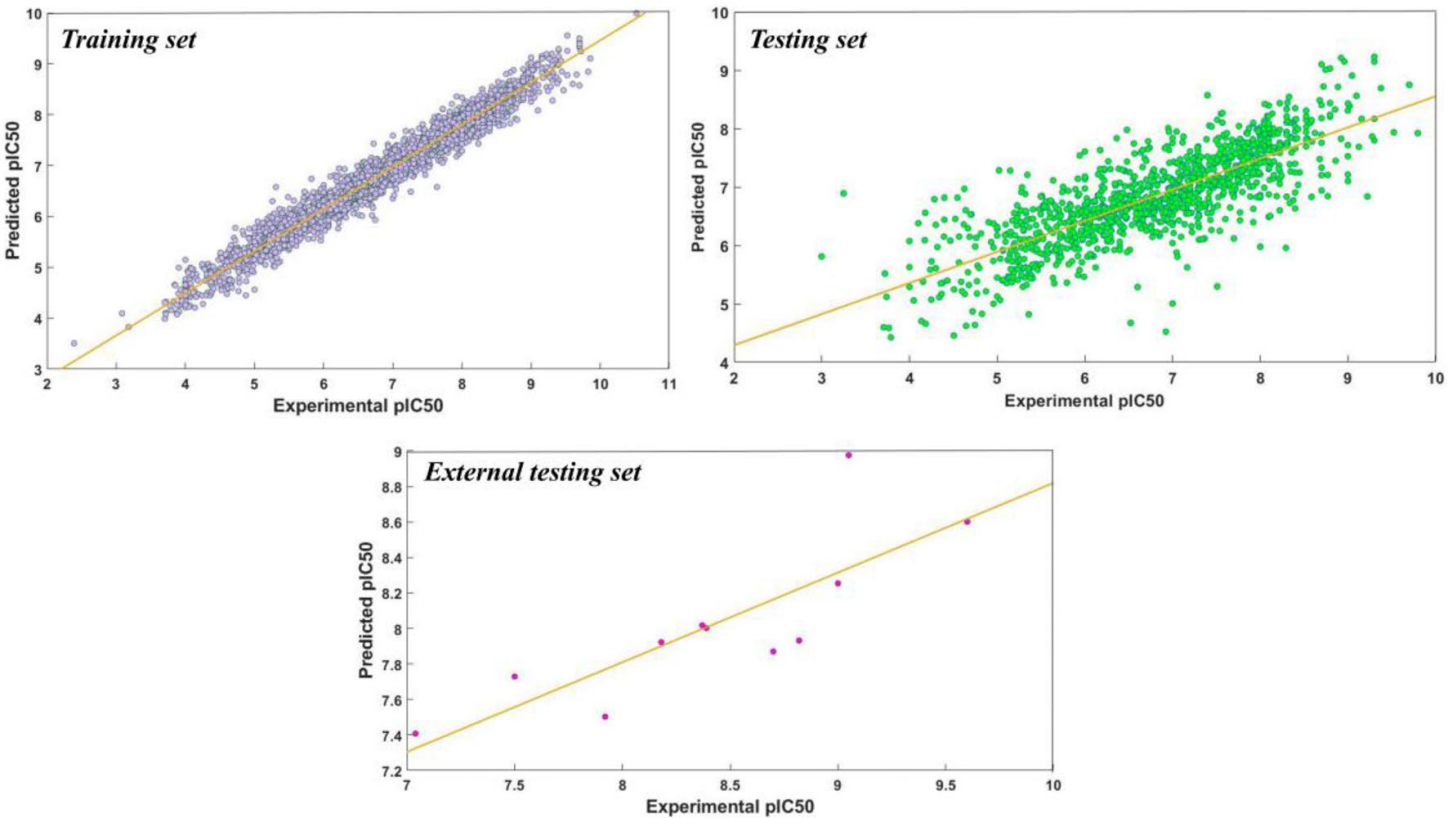
Experimental versus predicted pIC50 for training, testing, and external testing set. The orange line represents the data trendline. The figure is created by using WEKA 3.8.4 open-source software.

In addition to classification models, a regression model was employed to predict the pIC50 values of our candidate compounds. Fingerprints and molecular descriptors as the structural representative of compounds play a critical role in the QSAR model development and chemical information prediction. The 1024 bit-based ECFP of 5789 data is obtained using the alvaDesc tool to build the regression model^52,53^. The ECFP employs chemical information from the Daylight atomic invariants rule. These six properties include the valence minus the number of hydrogens, the atomic number, the atomic mass, the number of attached hydrogens, the atomic charge, and the number of non-hydrogen immediate neighbors^54^. Among statistical methods, such as multiple linear regression, k-nearest neighbor, multi-layer perceptron, SVM, and RF regression, the RF method as a popular algorithm is selected to build the predictive regression model because of its resistance to overfitting and ability to handle compounds with different mechanisms. It is also less time-consuming for variable selection. Another advantage of the RF method is the ability to perform the rapid type of cross-validation which is known as Out-of-Bag. The RF method also has shown excellent performance on a large number of data^55^. WEKA 3.8.4 software^56^ is used to apply the RF method to the current dataset. The dataset is split into 80% for training and 20% for the testing set. One thousand estimators are selected for the number of trees and the number of randomly chosen attributes, set as ${\varvec{i}}{\varvec{n}}{\varvec{t}}\left({{\varvec{l}}{\varvec{o}}{\varvec{g}}}_{2}\left(\#{\varvec{p}}{\varvec{r}}{\varvec{e}}{\varvec{d}}{\varvec{i}}{\varvec{c}}{\varvec{t}}{\varvec{o}}{\varvec{r}}{\varvec{s}}\right)+1\right)$. After fivefold cross-validation and out-of-bag estimates, the model was re-evaluated on the test set with 11 known VEGFR-2 inhibitors as an external test set as well to measure the performance of the model. The correlation coefficient, mean absolute error, and root-mean-squared error were obtained and are shown in Table 4. These criteria indicate that our model can predict biological activity with acceptable accuracy. The experimental versus predicted pIC50 values for training, testing, and external testing set are plotted in Fig. 6. Employing the above QSAR model, the pIC50 values for molecule1 and molecule2 are predicted to be 7.23 and 7.31, respectively which is comparable with the activity of pazopanib and sorafenib, which are 7.5 and 7.04, accordingly. The pIC50 values of some clinically approved VEGFR2 drugs are shown in Supplementary Table S1.

**Table 4:** The QSAR model performance on the training and testing dataset for VEGFR2 inhibitors. R2, Correlation coefficient; MAE, Mean absolute error; RMSE, (root-mean-squared error).

Statistical Metrics	Training	Testing	fivefold cross-validation	OOB	11 approved inhibitors
R^2^	0.9832	0.7879	0.7779	0.7959	0.8173
MAE	0.1992	0.5408	0.5583	0.5358	0.5044
RMSE	0.2655	0.7229	0.7386	0.7119	0.5831

### In-silico toxicity and ADMET prediction

ADMET prediction of potential drug compounds is an informative and important issue at the initial steps of drug discovery^57^. The SwissADME platform^58^ is applied to estimate the properties of drug candidates and regorafenib. Regorafenib, which has been approved by FDA for patients with hepatocellular carcinoma and metastatic colorectal cancer^59^, is used as a reference ligand for the ADMET prediction and MD simulations sections. The physicochemical properties, pharmacokinetics, drug-likeness, and medicinal chemistry of the molecules are shown in Supplementary Table S2. Molecule2 as a candidate for the anticancer agent is not a P-gp substrate, like what is predicted for regorafenib. Despite that, there are clinically approved drugs that are P-gp substrates^60^. The bioavailability score of 0.55 indicates the proper pharmacokinetic characteristics^57^. The percentage of absorption (%ABS) was estimated by %ABS = 109 − [0.345 × TPSA]^57,61,62^. %ABS values of regorafenib, molecule1, and molecule2 are ~ 77.1, 91.6, and 87.2, respectively, showing their significant permeability into the plasma membrane^57^. Molecule1 possesses high gastrointestinal (GI) absorption. Also, the log Kp values indicate the skin permeability ability of these compounds^57^ In addition, estimated Log S (ESOL)^63^ implies that regorafenib and molecule2 are moderately soluble in the body while molecule1 is poorly soluble. Additionally, the obtained bioavailability radar graphs provide a first glance regarding the drug likeness, as shown in Supplementary Figure S3. Each vertice shows the physicochemical properties, including lipophilicity, size, polarity, solubility, flexibility, and saturation^58^. The pink region displays the optimal region of drug-likeness. The drug-likeness properties for each molecule are represented by the red hexagon. In all cases, the saturation side is outside of the pink region, and in the case of molecule1, the LIPO vertice is slightly out of this region as well.

**Figure 7:**
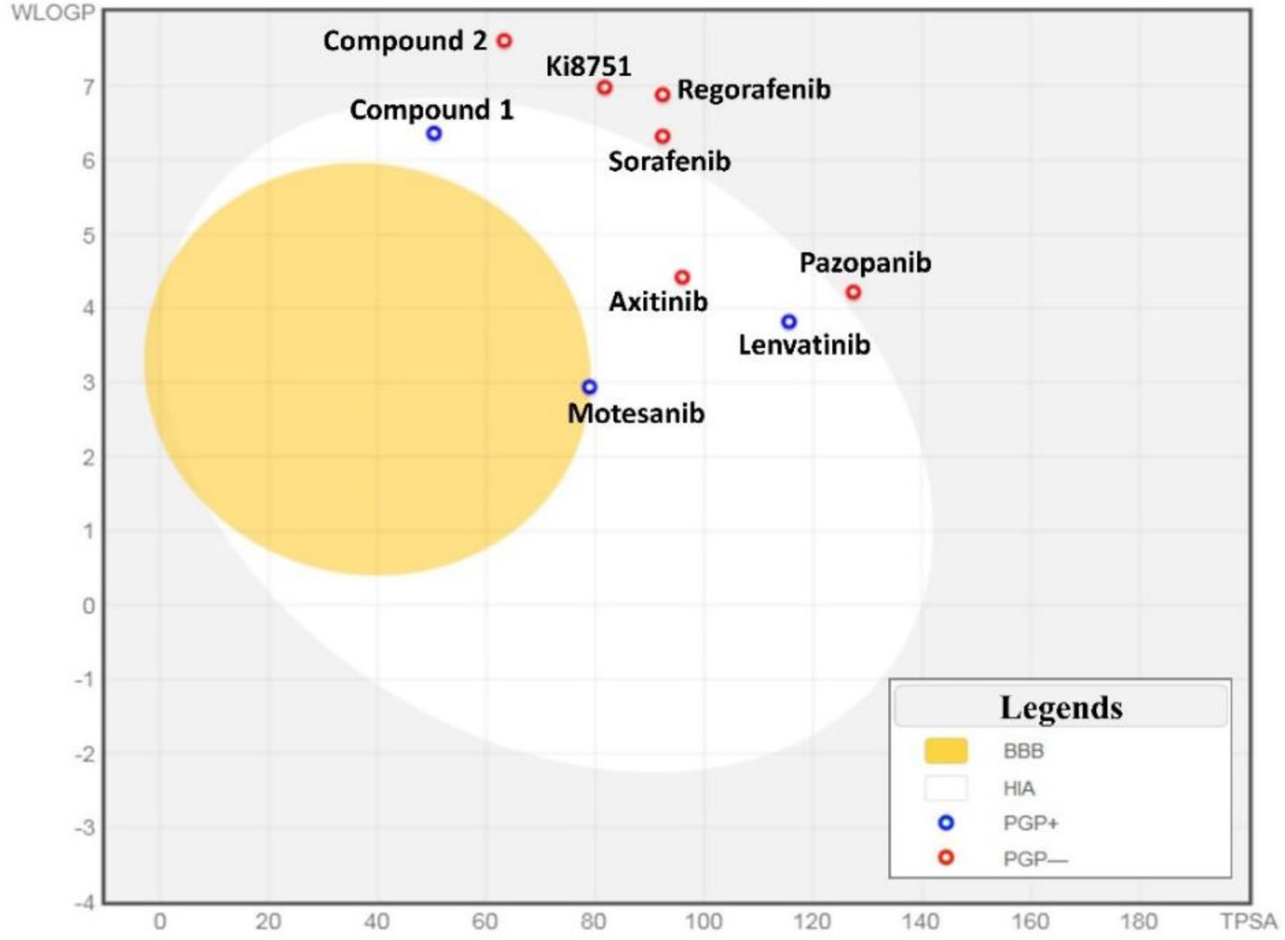
Predicted BOILED-Egg graph for intuitive evaluation of brain access (BBB) and gastrointestinal absorption (HIA) of 7 known inhibitors of VEGFR2 and 2 candidate compounds (molecule1 and molecule2). The result is based on lipophilicity (WLOGP) versus Topological polar surface area (TPSA). The figure is created by using SwissADME platform, www.swissadme.ch.

In addition, the Brain Or IntestinaL EstimateD permeation method (BOILED-Egg) is used to predict the permeation of the molecules. The BOILED-Egg model is based on the calculation of polarity and lipophilicity of molecules to estimate their passive human gastrointestinal absorption (HIA) and blood–brain barrier penetration (BBB)^64^. The obtained diagram is shown in Fig. 7. The yolk part is the physicochemical space for the molecules with the highest probability of brain penetration and the white part indicates highly probable GI absorption.

The molecules with predicted low HIA and BBB properties are shown in a gray zone. Also, the blue point shows the molecule is a substrate of P-gp and actively effluxed by P-gp, and the red point stands for the compound which is a non-substrate of P-gp. As depicted in Fig. 7 levatinib, axitinib, motesanib, and molecule1 predicted well-absorbed characteristics, however, only motesanib passively crossed the BBB but was pumped out from the brain. Other compounds were predicted to belong to the gray region which may imply a lower GI absorption^58,64,65^.

Moreover, The ProTox-II prediction tool^66^ which is based on chemical similarities is used to predict the median lethal dose (LD50), toxicity class, average similarity, and prediction accuracy^67^ of regorafenib as a reference drug and two candidate compounds. The oral toxicity prediction results are shown in Table 5. The predicted results indicated that these 3 compounds may belong to Class IV meanwhile molecule1 showed a higher LD50 value compared to regorafenib and molecule2, which could probably lead to less toxicity in this aspect. The maximum tolerated dose (MTD) for humans, which does not cause unacceptable side effects and can be used as a starting dose for clinical trials^68,69^, were calculated using the pkCSM tool^68^.

**Table 5:** Acute oral toxicity prediction using ProTox-II.

Chemical compound	Predicted LD50 (mg/kg)	Predicted toxicity class	Prediction accuracy (%)	Average Similarity (%)
Regorafenib	800	4	67.38	53.12
Compound 1	1600	4	69.26	75.56
Compound 2	393	4	68.07	67.42

The MTDs of regorafenib, molecule1, and molecule2 are obtained as 0.257, 0.527, and 0.428 (log mg/kg/day), respectively.

### Molecular docking and dynamics simulations of hit compounds

Docking and MD simulations were performed using the VEGFR2 crystal structure obtained from the protein data bank (PDB: 4ASE). Using Chimera software^30^ molecular docking was evaluated on seven drugs (pazopanib, sorafenib, axitinib, regorafenib, lenvatinib, motesanib, and Ki8751) and the two hit compounds (molecule1 and molecule2) to roughly estimate the strength of their bindings. The docking results indicated a good binding affinity between the candidate and the target protein compared to the interaction of known inhibitors. To obtain more accurate results on binding affinities, stability, and significant interactions, MD simulations were performed on molecule1, molecule2, and regorafenib for comparison. All MD simulations were performed using the GROMACS software. To ensure the convergence of the simulations, the root-mean-square deviation (RMSD) of the backbone and heavy atoms were calculated for protein and ligands, respectively. The RMSD plots for the whole trajectories are shown in the Supplementary Figure S2. As can be seen, the VEGFR2 complex with molecule1 and regorafenib were more stable in their specific pocket than the complex with molecule2. RMSDs for triplicates of all complexes were below 0.3 nm for the last 25 ns, which shows their stability^70^.

The complex including molecule1 became stable faster than the others. In the complex of molecule2, the protein was stable with low fluctuation while the ligand showed more fluctuation between 0.1 and 0.35 nm considering three trajectories. The RMSD values and visualization indicated that molecule2 mostly remained in the binging site and the fluctuation mainly occurred due to conformational changes in the allosteric hydrophobic pocket of the compound. Additionally, the low range of fluctuation and the RMSD value below around 0.3 nm for the last 25 ns indicated the stability of compound2 during the interaction. The obtained RMSDs for the full length of all trajectories revealed almost similar dynamics for each specific compound. Taken together these results and the low RMSD values implied that the MD simulation reached equilibrium and the compounds were stable in their binding sites. The ligand–protein binding free energies were calculated based on the Molecular Mechanics-Poisson Boltzmann Surface Area (MM-PBSA) approach over the last 25 ns of the equilibrated trajectories using the GROMACS software^71^. The binding free energies of molecule1, molecule2, and regorafenib complex with VEGFR-2 protein were − 89.1, − 95.3, and − 87.4 kJ/mol, respectively. Both molecule1 and molecule2 showed strong binding affinity and were comparable with the regorafenib binding free energy. The values of the energetic terms, displayed in Supplementary Table S3, revealed that Van der Waals interactions were the main contributor to the total binding free energy in all three cases, which was consistent with previous research that studied the interaction of some drugs with VEGFR2^72^.

**Figure 8:**
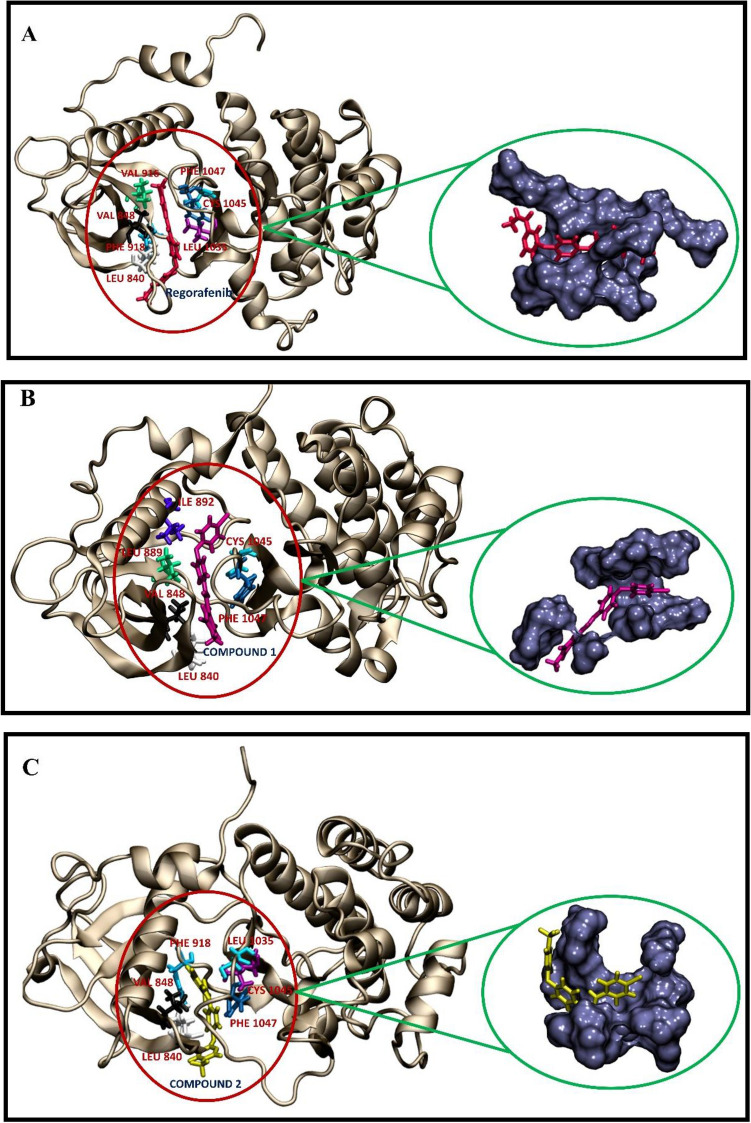
Geometries of residues that contributed highly to the binding free energy during the interaction between ligands and VEGFR2 crystal structure. The VEGFR2 structure was obtained from PDB ID: 4ASE. (**A**) Regorafenib-VEGFR2 complex. (**B**) Molecule1-VEGFR2 complex. (**C**) Molecule2-VEGFR2 complex. The residues and ligands are shown in stick form with different colors. The important residues are marked in red. The binding pockets within 3 Å surrounding the ligands were highlighted using the surface representation. The figures are created by using visual molecular dynamics (VMD) v.1.9.2, https://www.ks.uiuc.edu.

Figure 8 shows the residues of the protein that contributed more to the binding free energies and also that the binding pocket is located about 3 Å around the ligands. The highest binding interactions with the ligands in the VEGFR2-regorafenib, VEGFR2-compound, and VEGFR2-compound2 complexes were residues L840, V848, V916, F918, L1035, C1045, and F1047, residues L840, V848, L889, I892, V916, C1045, F1047, and residues L840, V848, F918, L1035, C1045, F1047, respectively. Residues L840, V848, C1045, and F1047 were common in all three cases. Additionally, these key amino acids are highly hydrophobic residues that implied the primary contribution of Van der Waals forces in the binding energies. The pivotal role of hydrophobic interactions in VEGFR2 inhibition was shown in other studies^72,73^.

The above results indicated that the binding strength of the candidate compounds to the binding pocket of the target protein could be almost the same or even higher than the regorafenib, an approved drug, which may make them useful as inhibitors. The details of the interaction mechanism between VEGFR2 proteins and inhibitors can provide critical information for potent drugs in the future as well.

## Conclusions

In this research study, we constructed a workflow based on ligand-based virtual screening integrated with similarity measures with some clinically approved drugs to find potential inhibitors that could target the signaling pathway of the VEGFR2 tyrosine kinase. The RF model was selected as the best model for classification to predict the probability of activity. Using a large external library (~ 10,500,000 molecules) for classification and similarity checking with available drugs, 2 compounds (molecule1 (PubChem CID 17379777 and molecule2 (PubChem CID 4682044)) were selected as the final candidates for further analysis. Using the RF regression approach the pIC50 values of the two candidate compounds were predicted to be ~ 7.23 and ~ 7.31 which was comparable with the inhibition activity of the drugs pazopanib and sorafenib. In addition, ADMET analysis, molecular docking simulations, and MD simulations were performed on the two candidates, with regorafenib as a reference drug. The predicted LD50 values for molecule1, molecule2, and regorafenib were 1600, 393, and 800 mg/kg, respectively. Results from docking characterizations and MD simulations conducted on known drugs and the two new compounds revealed the high affinity between the two compounds and VEGFR2, which was comparable with the regorafenib interaction. The binding free energy for molecule1, molecule2, and regorafenib were -89.1, -95.3, and -87.4 kJ/mol, respectively. Notably, the detailed analysis emphasized the significance of hydrophobic interactions in the VEGFR2 inhibition process. Our workflow and analysis may provide valuable information for anti-angiogenesis therapy and the two introduced inhibitor candidates could be further investigated as potent anti-cancer agents.

## Supplementary Information

Supplementary Information.

## Data Availability

All the PDB files were obtained from the RCSB protein data bank (http://www.rcsb.org/). The GROMACS v.5.0 (developed at the University of Groningen), visual molecular dynamics (VMD) v.1.9.2 (developed at the University of Illinois at Urbana-Champaign), UCSF Chimera v.1.12 (developed at the University of California), and WEKA 3.8.4 open-source software were employed for simulation and calculations. OpenCADD, SwissADME, and ProTox-II free platforms were used in this study as well. The data and results are included in this article and the supporting information.

## Abbreviations

ML: Machine learning
ANN: Artificial neural network
RF: Random forest
NB: Naïve Bayesian
SVM: Support vector machine
VEGFR: Vascular endothelial growth factor receptor
RMSD: Root-mean-square deviation
MAE: Mean absolute error
RMSE: Root-mean-squared error
AUC: Area under the curve
