# Supplementary material for: The use of machine learning modeling, virtual screening, molecular docking, and molecular dynamics simulations to identify potential VEGFR2 kinase inhibitors
Source: Sci Rep. 2022 Nov 5;12:18825. doi: 10.1038/s41598-022-22992-6 (PMC9637137; doi:10.1038/s41598-022-22992-6)
Supplement: Supplementary file 1 — Supplementary Information. [file 41598_2022_22992_MOESM1_ESM.pdf]

Supplementary Information

The use of machine learning modeling, virtual screening, molecular docking, and molecular dynamics simulations to identify potential VEGFR2 kinase inhibitors

Abbas Salimi[‡], *Jong Hyeon Lim*[‡], *Jee Hwan Jang*[*,†,#] *and Jin Yong Lee*[*,‡]

[‡] Department of Chemistry, Sungkyunkwan University, Suwon 16419, Korea;

[†] School of Materials Science and Engineering, Sungkyunkwan University, Suwon 16419, Korea;

[#] Ucaretron Inc., No. 3508, 40, Simin-daero 365 beon-gil, Dongan-gu, Anyang-si, Gyeonggi-do, Korea;

Figure.S1. Similarity checking for Pazopanib, Sorafenib, Axitinib, Regorafenib, Lenvatinib, Motesanib, and Ki8751 with top-ranked molecules applying the Morgan2 and MACCS fingerprints

Table.S1. The pIC50 values of some clinically approved VEGFR2 drugs

Figure.S2. Root mean square deviation (RMSD) of A) Compound1-VEGFR2, B) Compound2-VEGFR2, and C) Regorafenib-VEGFR2 complex. (The MD simulations were performed in triplicates for each case)

Table.S2. Physicochemical, pharmacokinetics, drug-likeness, and medicinal properties of regorafenib, molecule1 and 2 predicted by SwissADME tool.

Table.S3. Average Van der Waals, electrostatic, polar solvation, SASA (solvent accessible surface area), and total binding free energy (standard error). All units are in kJ/mol.

Figure.S3. The Bioavailability Radar (pink represents the optimal range for each property. LIPO: XLOGP3 between −0.7 and +5.0, SIZE: Molecular weight between 150 and 500 g/mol, POLAR: TPSA between 20 and 130 Å2, INSOLU: log S not higher than 6, INSATU: fraction of carbons in the sp3 hybridization not less than 0.25, and FLEX: no more than 9 rotatable bonds. A) Regorafenib. B) Molecule1. C) Molecule2.

1) Pazopanib

	name	tanimoto_morgan	dice_morgan	tanimoto_maccs	dice_maccs
0	0	1.000000	1.000000	1.000000	1.000000
1	29	0.235294	0.380952	0.796875	0.886957
2	3	0.207792	0.344086	0.777778	0.875000
3	12	0.207317	0.343434	0.781250	0.877193
4	50	0.185185	0.312500	0.363636	0.533333
...	...	...	...	...	...
56	19	0.107143	0.193548	0.314286	0.478261
57	48	0.105882	0.191489	0.314286	0.478261
58	51	0.103448	0.187500	0.352113	0.520833
59	54	0.082474	0.152381	0.329114	0.495238
60	52	0.073684	0.137255	0.342105	0.509804

2) Sorafenib

	name	tanimoto_morgan	dice_morgan	tanimoto_maccs	dice_maccs
0	0	1.000000	1.000000	1.000000	1.000000
1	40	0.446154	0.617021	0.854167	0.921348
2	16	0.433333	0.604651	0.729167	0.843373
3	37	0.232877	0.377778	0.560000	0.717949
4	39	0.230769	0.375000	0.523077	0.686869
...	...	...	...	...	...
56	9	0.095890	0.175000	0.345455	0.513514
57	8	0.093333	0.170732	0.375000	0.545455
58	28	0.083333	0.153846	0.327273	0.493151
59	52	0.066667	0.125000	0.294118	0.454545
60	54	0.064516	0.121212	0.263889	0.417582

3) Axitinib

	name	tanimoto_morgan	dice_morgan	tanimoto_maccs	dice_maccs
0	0	1.000000	1.000000	1.000000	1.000000
1	38	0.232877	0.377778	0.509804	0.675325
2	23	0.226667	0.369565	0.750000	0.857143
3	20	0.225352	0.367816	0.461538	0.631579
4	19	0.222222	0.363636	0.500000	0.666667
...	...	...	...	...	...
56	8	0.106667	0.192771	0.416667	0.588235
57	2	0.106667	0.192771	0.416667	0.588235
58	35	0.102564	0.186047	0.408163	0.579710
59	52	0.102273	0.185567	0.362069	0.531646
60	24	0.101124	0.183673	0.430769	0.602151

4) Regorafenib

	name	tanimoto_morgan	dice_morgan	tanimoto_maccs	dice_maccs
0	0	1.000000	1.000000	1.000000	1.000000
1	40	0.369863	0.540000	0.854167	0.921348
2	16	0.352941	0.521739	0.729167	0.843373
3	56	0.240506	0.387755	0.580000	0.734177
4	37	0.230769	0.375000	0.560000	0.717949
...	...	...	...	...	...
56	9	0.088608	0.162791	0.345455	0.513514
57	8	0.086420	0.159091	0.375000	0.545455
58	28	0.076923	0.142857	0.327273	0.493151
59	52	0.062500	0.117647	0.294118	0.454545
60	54	0.060606	0.114286	0.263889	0.417582

5) Lenvatinib

	name	tanimoto_morgan	dice_morgan	tanimoto_maccs	dice_maccs
0	0	1.000000	1.000000	1.000000	1.000000
1	40	0.243902	0.392157	0.684211	0.812500
2	16	0.236842	0.382979	0.636364	0.777778
3	43	0.209877	0.346939	0.586207	0.739130
4	33	0.209877	0.346939	0.517857	0.682353
...	...	...	...	...	...
56	8	0.097561	0.177778	0.354839	0.523810
57	2	0.097561	0.177778	0.354839	0.523810
58	52	0.094737	0.173077	0.338028	0.505263
59	35	0.094118	0.172043	0.349206	0.517647
60	54	0.091837	0.168224	0.324324	0.489796

6) Motesanib

	name	tanimoto_morgan	dice_morgan	tanimoto_maccs	dice_maccs
0	0	1.000000	1.000000	1.000000	1.000000
1	46	0.305556	0.468085	0.744681	0.853659
2	38	0.295775	0.456522	0.555556	0.714286
3	42	0.263889	0.417582	0.574468	0.729730
4	49	0.260870	0.413793	0.510638	0.676056
...	...	...	...	...	...
56	2	0.118421	0.211765	0.388889	0.560000
57	8	0.118421	0.211765	0.388889	0.560000
58	52	0.112360	0.202020	0.457627	0.627907
59	24	0.111111	0.200000	0.351351	0.520000
60	28	0.109589	0.197531	0.420000	0.591549

7) Ki8751

	name	tanimoto_morgan	dice_morgan	tanimoto_maccs	dice_maccs
**0**	0	1.000000	1.000000	1.000000	1.000000
**1**	40	0.260274	0.413043	0.760000	0.863636
**2**	56	0.250000	0.400000	0.560000	0.717949
**3**	55	0.235294	0.380952	0.530612	0.693333
**4**	16	0.235294	0.380952	0.708333	0.829268
**...**	...	...	...	...	...
**56**	8	0.095890	0.175000	0.407407	0.578947
**57**	21	0.095890	0.175000	0.407407	0.578947
**58**	24	0.091954	0.168421	0.383562	0.554455
**59**	52	0.080460	0.148936	0.279412	0.436782
**60**	54	0.077778	0.144330	0.267606	0.422222

Figure.S1. Similarity checking for Pazopanib, Sorafenib, Axitinib, Regorafenib, Lenvatinib, Motesanib, and Ki8751 with top-ranked molecules applying the Morgan2 and MACCS fingerprints

Table.S1. The pIC50 values of some clinically approved VEGFR2 drugs

Inhibitors	pIC50
Pazopanib	7.5
Sorafenib	7.04
axitinib	9.6
Regorafenib	8.37
Lenvatinib	8.39
Motesanib	8.7
Ki8751	9.05

A

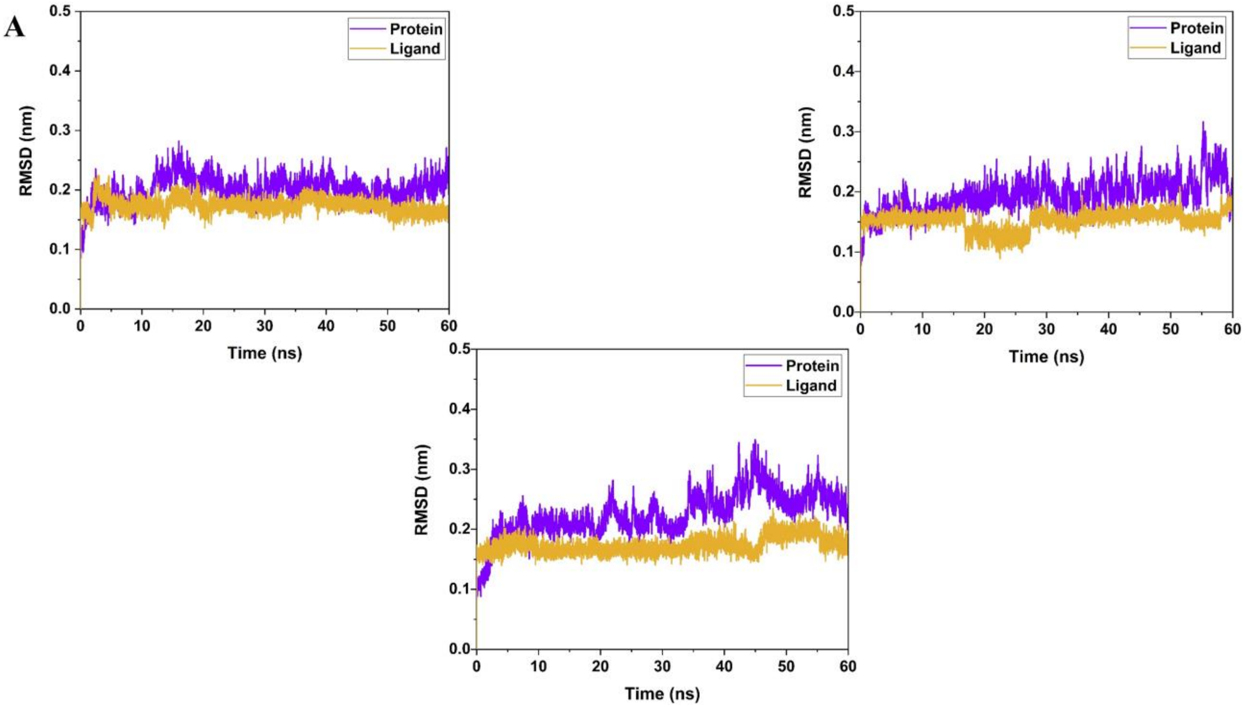

Protein
Ligand
RMSD (nm)
Time (ns)
0.0
0.1
0.2
0.3
0.4
0.5
0
10
20
30
40
50
60

B

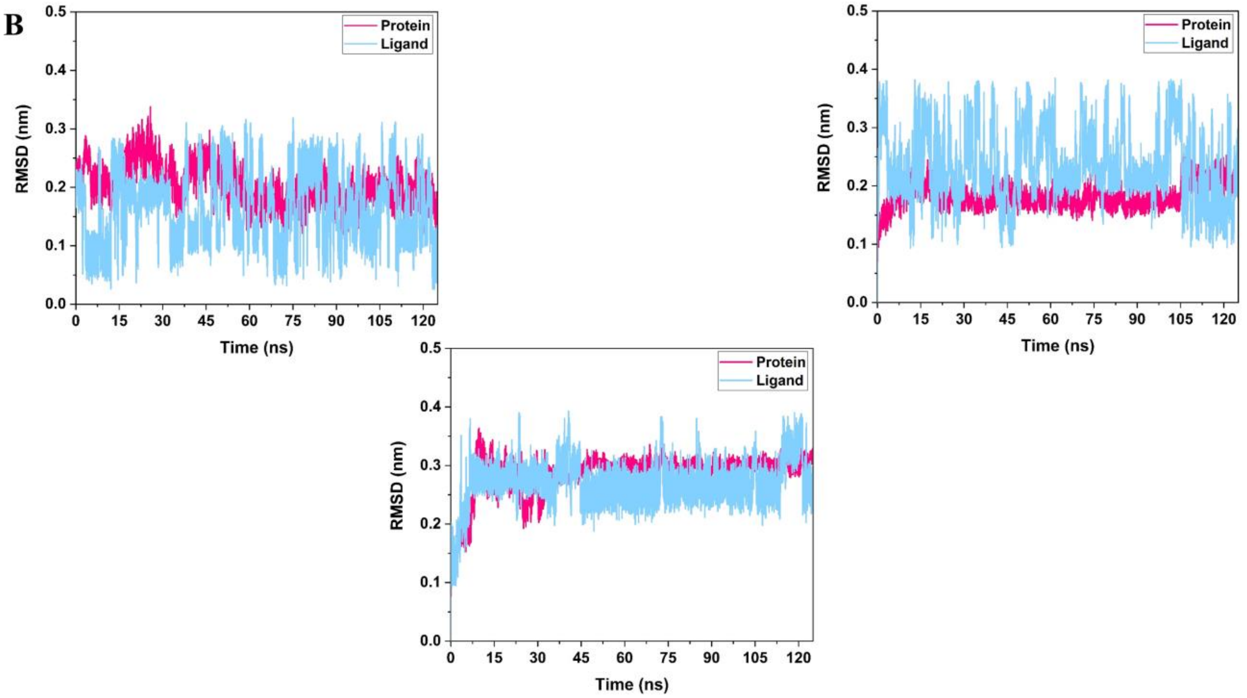

Protein
Ligand
RMSD (nm)
Time (ns)
0.0
0.1
0.2
0.3
0.4
0.5
0
15
30
45
60
75
90
105
120

C

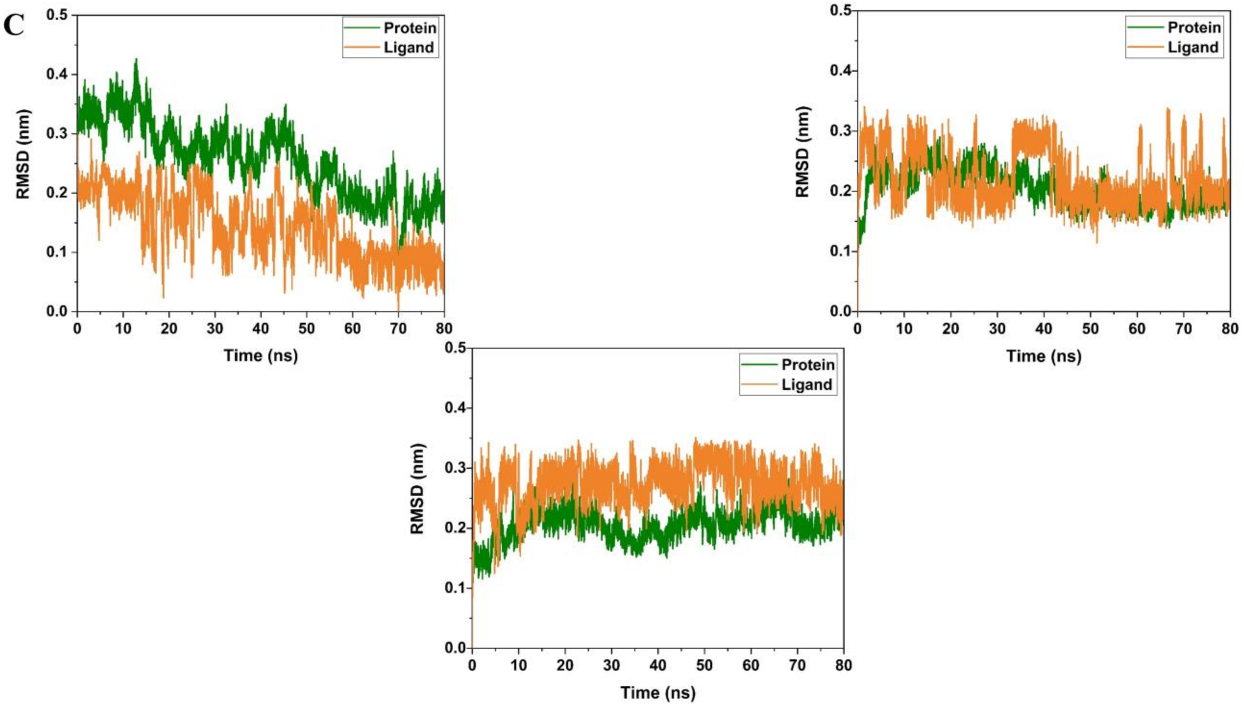

Figure.S2. Root mean square deviation (RMSD) of A) Compound1-VEGFR2, B) Compound2-VEGFR2, and C) Regorafenib-VEGFR2 complex. (The MD simulations were performed in triplicates for each case)

Table.S2. Physicochemical, pharmacokinetics, drug-likeness, and medicinal properties of regorafenib, molecule1 and 2 predicted by SwissADME tool.

Compound	Physicochemical Properties		Pharmacokinetics		Druglikeness		Medicinal Chemistry	
**Regorafenib**	**Molecular weight (g/mol)**	482.82	**GI absorption**	Low	**Lipinski**	Yes; 0 violation	**PAINS**	0 alert
	**Num.heavy atoms**	33	**BBB permeant**	No	**Ghose**	No; 2	**Brenk**	0 alert
	**Num.arom. heavy atoms**	18	**P-gp substrate**	No	**Veber**	Yes	**Leadlikeness**	No; 3
	**Fraction Csp3**	0.10	**CYP1A2 inhibitor**	Yes	**Egan**	No; 1	**Synthetic accessibility**	3.04
	**Num.rotatable bonds**	9	**CYP2C19 inhibitor**	Yes	**Muegge**	Yes		
	**Num.H-bond acceptors**	8	**CYP2C9 inhibitor**	Yes	**Bioavailability Score**	0.55		
	**Num.H-bond donors**	3	**CYP2D6 inhibitor**	Yes				
	**MolarRefractivity**	112.44	**CYP3A4 inhibitor**	Yes				
	**TPSA (Å²)**	92.35	**Log Kp (skin permeation) (cm/s)**	-6.28				
**Molecule 1**	**Molecular weight (g/mol)**	387.26	**GI absorption**	High	**Lipinski**	Yes; 1	**PAINS**	0 alert
	**Num.heavy atoms**	26	**BBB permeant**	No	**Ghose**	No; 1	**Brenk**	0 alert
	**Num.arom. heavy atoms**	18	**P-gp substrate**	Yes	**Veber**	Yes	**Leadlikeness**	No; 2
	**Fraction Csp3**	0.05	**CYP1A2 inhibitor**	No	**Egan**	No; 1	**Synthetic accessibility**	2.37
	**Num.rotatable bonds**	6	**CYP2C19 inhibitor**	Yes	**Muegge**	No;1		
	**Num.H-bond acceptors**	2	**CYP2C9 inhibitor**	Yes	**Bioavailability Score**	0.55		
	**Num.H-bond donors**	2	**CYP2D6 inhibitor**	Yes				
	**MolarRefractivity**	106.67	**CYP3A4 inhibitor**	No				
	**TPSA (Å²)**	50.36	**Log Kp (skin permeation) (cm/s)**	-4.24				
**Molecule 2**	**Molecular weight (g/mol)**	442.22	**GI absorption**	Low	**Lipinski**	Yes; 1	**PAINS**	0 alert
	**Num.heavy atoms**	29	**BBB permeant**	No	**Ghose**	No;1	**Brenk**	0 alert
	**Num.arom. heavyatoms**	18	**P-gp substrate**	No	**Veber**	Yes	**Leadlikeness**	No; 2
	**Fraction Csp3**	0.05	**CYP1A2 inhibitor**	Yes	**Egan**	No; 1	**Synthetic accessibility**	2.83
	**Num.rotatable bonds**	7	**CYP2C19 inhibitor**	Yes	**Muegge**	Yes		
	**Num.H-bond acceptors**	6	**CYP2C9 inhibitor**	Yes	**Bioavailability Score**	0.55		
	**Num.H-bond donors**	2	**CYP2D6 inhibitor**	Yes				
	**MolarRefractivity**	104.50	**CYP3A4 inhibitor**	Yes				
	**TPSA (Å²)**	63.25	**Log Kp (skin permeation) (cm/s)**	-5.47				

Table.S3. Average Van der Waals, electrostatic, polar solvation, SASA (solvent accessible surface area), and total binding free energy (standard error). All units are in kJ/mol.

Complex	van der Waals	Electrostattic	Polar solvation	SASA	Total Binding
**VEGFR2-Compound1**	-192.899 (0.192)	-14.746 (0.112)	139.669 (0.285)	-21.112 (0.018)	-89.074 (0.294)
**VEGFR2-Compound2**	-168.380 (0.327)	-24.69 (0.147)	114.073 (0.334)	-16.308 (0.027)	-95.326 (0.275)
**VEGFR2-Regorafenib**	-220.938 (0.258)	-39.481(0.247)	195.036 (0.569)	-22.014 (0.022)	-87.384 (0.327)

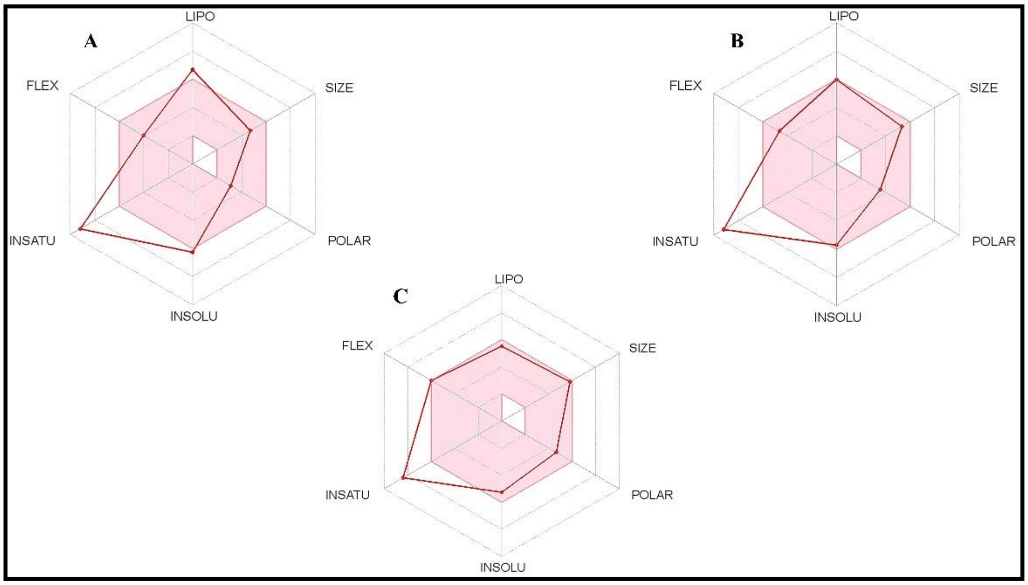

Figure.S3. The Bioavailability Radar (pink represents the optimal range for each property. LIPO: XLOGP3 between −0.7 and +5.0, SIZE: Molecular weight between 150 and 500 g/mol, POLAR: TPSA between 20 and 130 Å2, INSOLU: log S not higher than 6, INSATU: fraction of carbons in the sp3 hybridization not less than 0.25, and FLEX: no more than 9 rotatable bonds. A) Regorafenib. B) Molecule1. C) Molecule2.
